# Supplementary material for: Are carbon emissions trading and green financial instruments synergistic? -Comprehensive quantitative research based on content analysis
Source: PLoS One. 2024 Mar 7;19(3):e0298601. doi: 10.1371/journal.pone.0298601 (PMC10919601; doi:10.1371/journal.pone.0298601)
Supplement: S1 Data — (ZIP) [file pone.0298601.s001.zip › Data/地级市Z_F工作报告.zip!/║┌┴·╜¡/╞δ╞δ╣■╢√╩╨/└┤╫╘╣1⁄2╓┌║┼í╛┬φ┐╦╩2╛▌═°í┐_2018.docx]

各位代表：

　　现在，我代表市人民政府，向大会报告工作，请予审议，并请各位政协委员提出意见。

　　2017年，在市委的领导下，我们坚持以习近平总书记系列重要讲话精神为指引，贯彻新发展理念，围绕做好“三篇大文章”，加快构建新型产业体系，实施重点产业倍增计划，推动全面振兴发展，经济社会发展态势持续向好。预计全市地区生产总值同比增长6%以上；固定资产投资增长7%以上；公共财政预算收入按可比口径增长4.7%，其中地方税收增长7.3%；城乡居民人均可支配收入分别增长7%和6.5%左右；万元GDP综合能耗下降3.5%；主要污染物减排完成省下达计划。

　　——农业产业化进程加快。粮食总产实现恢复性增长,农业增加值预计增长5%以上。坚持市场导向调整种植结构，三大作物呈现“一减两增”的结构变化，新增杂粮杂豆、蔬菜、中草药等经济作物94万亩、食用菌近7000万袋。新建生态高产标准农田113万亩，建设“互联网+农业”省级示范基地71万亩，新增节水灌溉4万亩。推动畜牧业规模化发展，建设标准化养殖场65个，其中新建百万头生猪养殖基地5个;生猪出栏580万头，高档肉牛饲养量达到10万头，奶牛标准化规模养殖承载能力达到22万头。新增省级农民合作社规范社25个、联合社20个，新增省市级农产品龙头企业47户，一批涉农企业向“储加销”农业公司转型。组织7场（次）农产品大型外埠集中推广营销。飞鹤奶山羊养殖及乳品加工、雪川马铃薯全产业链等项目加快建设，鸿展食用酒精项目建设完工，阜丰玉米深加工项目一期当年开工当年投产，单体投资规模和建设速度实现历史性突破。

　　——工业经济恢复增长。规上工业增加值预计增长6%以上。装备制造业在一重、齐车等骨干企业强力拉动下加快复苏，食品加工业在乳品行业支撑下平稳运行。“五化”示范企业达到10户，纳入省级数字化示范车间企业6户，省重点领域首台套产品6项。齐车重载快捷货车制造项目建成投产，华鹤数字化制造系统上线运行，华工机床大力矩电机实现产业化。新增规上企业27户，央企地方配套率达到35%。推动企业降本增效，贷款周转金、助保金贷款分别增长11.8%和17.4%；直供电企业达到39户、节省支出6500多万元；失业保险缴费率下调至0.5%；减停一批涉企收费，为企业减负1.1亿元。紫金铜产业园开工建设，甘南工业示范基地晋升为省级经济开发区。

　　——服务业稳步发展。社会消费品零售总额预计增长9%左右。新增限上企业26户，限上企业触网率52%。电商企业发展到232家，自建电商平台25个、园区12个，网商微商超过5万个，电商上行销售额突破20亿元、增长30%以上。市区农信社改制获得批筹，完成克东农商行改制，成立讷河村镇银行，新增新三板和区域股权交易市场挂牌企业15户。金融机构贷款余额稳定在1500亿元以上。扎龙旅游带、碾子山全域旅游等项目扎实推进，新增国家级旅游示范基地2个，4A级景区达到10家，加强系统化集成化推广营销，接待旅游人数和总收入分别增长10%和20%。设立全国首个城市冰球节，举办冬夏两个冰球季，承办国际国内冰雪运动赛事100余场次，黑龙冰刀智能化生产线和台湾八达雪橇项目建成投产。推出话剧《萧红》、冰上杂技《飞雪迎春》等精品剧目，商演300余场次。创办嫩江玛瑙电商基地，建立工艺陶瓷大师工作室12个。市失能老人康复中心等13个养老机构投入运营，新增床位2000余张。

　　——改革创新不断深入。统筹推进“放管服”改革，精简市级行政权力891项，差异化向区下放165项，启动实施“多规合一”“多审合一”“承诺制、零审批”、自然资源统一确权登记制度改革试点，深化公共资源集中交易、城市管理综合执法改革，社会信用体系建设和“多证合一”等商事登记制度改革加快推进，新增市场主体4万余户。政务云建设全面启动，市区“一窗式”政务服务平台投入运行。全面建立市县（区）新的共享税体制。公立医院改革全面推开，事业单位分类等改革有序推进。北满特钢成功重整运营，完成一厂医院改制，推动富钢改制生成新项目，厂办大集体改革、农村土地确权实测基本完成。生成科技成果296项，域外科技成果落地50项，新增授权专利1200余项。新增重点实验室、企业技术中心、院士工作站等科研机构14个，新建科技企业孵化器18个，高新区孵化器启动运营，组建国家洁净钢工艺与检测创新技术产业联盟和省金属新材料产业技术创新战略联盟，成为工信部G7高校联盟论坛固定举办城市。新增国家高新技术企业6户。三间房国际经贸物流产业园启动建设，进口肉类指定查验场获国家批筹，齐欧冷链专列开通。与广州市对口合作全面启动。

　　——民生持续改善。系统实施“七大民生工程”，全市民生支出增长13.8%。扎实开展精准扶贫“回头看”，落实“两不愁、三保障”政策，完善“三有三通”基础设施，投入各类扶贫资金近30亿元，252个贫困村、5.81万人实现脱贫，贫困发生率降至2.23%。发放创业担保贷款1.5亿元、就业补贴4.5亿元、援企稳岗补贴4400多万元，创业平台达到183个，城镇新增就业再就业近10万人，城镇登记失业率控制在4.31%。城镇企业退休职工、城乡居民养老金月人均分别提高137元和10元，并按时足额发放。失业保险待遇提高10%。城乡居民医保住院报销比例提高5%，最高支付限额提高3万元，开展长期护理保险试点，“四类人群”实现异地就医直接结算。城乡低保标准年人均分别提高到6480元和3780元，发放各类救助资金 10.3亿元。建成棚改房1.87万套，改造农村泥草危房2.42万户。中心城区综合改造老旧小区16个，解决了248栋单散楼物业区域整合和366栋弃管楼物业服务问题。浏园水厂扩建项目启动，铺设供水管线74.2公里，改造老旧供热管网11公里。城区更新纯电动公交车852台，新建公交首末站16处，施划停车位6.4万个。升级改造市政道路121条，中心城区东北部环路贯通。北富高速等4条公路建成通车，修建农村公路730公里。高标准完成国家节能减排示范市创建任务。淘汰燃煤小锅炉570座、黄标车1万余台。全面实施河长制，强化饮用水源地保护和黑臭水体整治，启动氧化塘水生态修复工程，全市地表水Ⅲ类以上水体比例提高16.7%。启动畜禽粪污专项整治。化肥、农药、除草剂施用量减少5%以上。实施“四季环境整治”，拆除违章建筑1.8万处，植树造林9.5万亩。改造薄弱学校79所，建设标准化学校46所，3个区通过国家义务教育基本均衡县（区）验收。重点人群家庭医生签约率达到67%，建立名医工作室12个。新增全民健身路径550处。恢复举办龙沙音乐会，文化惠民演出240场，启动卜奎清真寺等重点文保修缮项目。深化“平安鹤城”建设，加强社会治安防控，发案下降27%；食药品安全形势稳定向好，生产安全事故持续下降；一批信访积案有效化解。新建城乡示范社区25个。圆满完成“十九大”期间安保维稳任务。

　　——政府自身建设扎实推进。认真学习贯彻党的十九大精神，深入推进“两学一做”学习教育常态化制度化。全面推进法治政府建设，基本形成市县乡三级法律顾问工作体系。自觉接受人大依法监督、政协民主监督和社会监督，办理人大意见建议162件、政协提案300件。“双随机、一公开”执法机制全面推开，行政许可和行政处罚信息“双公示”全覆盖。落实党风廉政建设责任制，加强行政监察和审计监督。扎实开展机关作风整顿，查处各类破坏发展环境案件152件。规范完善政府投资专项预算管控，一般性支出压缩5%。

　　社会科学、民族宗教、新闻出版、妇女儿童、机关事务、地方志、统计、物价、档案、人防、双拥、老龄、残联、气象、地震等工作，也都取得了新成绩。

　　各位代表！这一年，我们在转型发展任务繁重、改革进入攻坚期的环境下，取得来之不易的成绩，得益于习近平新时代中国特色社会主义思想和习近平总书记对我省重要讲话精神的正确指引，得益于省委、省政府和市委的坚强领导，得益于市人大、市政协的监督支持，得益于全市广大干部群众的团结奋进。在此，我代表市人民政府，向全市人民，向各级人大代表、政协委员，向各民主党派、工商联、无党派人士和人民团体，向驻齐解放军、武警官兵和中省直单位，向所有关心支持齐齐哈尔振兴发展的各界人士，表示诚挚的感谢！

　　在总结回顾发展变化和取得成绩的同时，我们也清醒地看到存在的差距和不足。在经济领域方面，农业适应市场化和抵御灾害能力较弱，种植结构不稳定，畜牧产业链不健全，新型经营主体培育不足，农产品价值优势释放不充分；工业内部分化趋势明显，机制、模式和产品创新不够，地方配套率较低，产能释放不足，核心竞争力有待提升；服务业电商融合化发展不快，稳固域内市场压力较大，辐射域外市场能力不强，进出口贸易和金融服务支持实体经济仍有很大空间。在民生领域方面，就业岗位开发不多、创业氛围不浓，社会保障力仍需提高，看病难看病贵尚未根本解决，污染整治、生态保护任务依然艰巨，在物业服务、供暖质量、城市交通、教育发展等方面，与群众的需要还有很大差距。在政府自身建设方面，改革的定力和勇气不足，深耕效果不明显，创新的热情和能力不高，作风不实、效率不高、服务不优问题依然存在，一些领域和部门的假把式、软把式、歪把式仍很突出。对这些问题，我们一定高度重视，认真加以解决。

　　2018年是贯彻党的十九大精神的开局之年，是决胜全面建成小康社会、实施“十三五”规划承上启下的关键一年。我国经济发展进入了新时代，经济由高速增长阶段转向高质量发展阶段。我市发展也进入了决胜全面建成小康社会的新时期和推动全面振兴发展的重要关口。同时，随着我国社会主要矛盾的转化，我市发展不平衡不充分的问题更加凸显。但从总体上判断，机遇大于挑战。只要以新的历史方位和时代坐标审视我市发展，牢牢把握国家新一轮东北等老工业基地振兴、“中国制造2025”“一带一路”等战略机遇，提振信心，奋力前行，就一定能在建设富强民主文明和谐美丽新鹤城的征程中，谱写新篇章，再创新辉煌！

　　政府工作的总体思路是：高举中国特色社会主义伟大旗帜，全面贯彻党的十九大精神，以习近平新时代中国特色社会主义思想为指导，深入落实中央经济工作会议、省委十二届二次全会和市委十三届二次全会精神，坚持稳中求进工作总基调，坚持新发展理念，紧扣社会主要矛盾变化，按照高质量发展的要求，统筹推进“五位一体”总体布局和协调推进“四个全面”战略布局，坚持以供给侧结构性改革为主线，统筹推进稳增长、促改革、调结构、惠民生、防风险各项工作，加快建设工业强市、农业强市、科教强市、文化强市、生态强市、旅游强市，坚决打好转方式调结构、精准脱贫、重要领域改革、污染防治、防范化解重大风险、整顿作风优化发展环境攻坚战，突出改革创新，着力构建以12个重点产业为支撑的现代产业体系，着力保障和改善民生，着力建设生态文明，着力完善社会治理体系，着力加强政府自身建设，为建设富强民主文明和谐美丽新鹤城奠定坚实基础。

　　主要预期目标是：全市地区生产总值增长6.5%左右，规上工业增加值增长6.5%左右，固定资产投资增长5%左右，公共财政预算收入增长2%以上，社会消费品零售总额增长8%左右，城乡居民人均可支配收入分别增长7%和6.5%以上，城镇登记失业率控制在4.3%，万元GDP综合能耗下降3.5%，主要污染物减排任务全面完成。

　　为完成上述目标，重点做好以下工作：

　　一、坚持深化供给侧结构性改革，大力培育市场主体，加快构建现代产业体系

　　把发展着力点放在实体经济上，把提高供给体系质量作为主攻方向，把培育市场主体作为重要抓手，加快建设“四个协同”发展的产业体系，推动12个重点产业提质增效，打好转方式调结构攻坚战。

　　（一）以实施乡村振兴战略为统领，着力培育新型农业经营主体，加快建设农业强市。按照“产业兴旺、生态宜居、乡风文明、治理有效、生活富裕”总要求，科学制定乡村振兴规划，加快构建现代农业“三大体系”，促进一二三产业融合发展，推进质量兴农、绿色兴农。围绕市场需求增加优质农产品供给。坚持优品质，按照市场需求合理调整玉米、水稻、大豆、杂粮种植面积，增加中草药、食用菌、蔬菜等高效经济作物。完成国家粮食生产功能区和重要农产品生产保护区划定任务。加快生态高产标准农田建设，启动实施农田水利建设五年提升工程。按照“稳两牛、扩生猪、适度发展羊、特色发展禽”的思路，推进标准化规模养殖，提高养殖质量、效益和供给力。加快东方希望、牧原、大北农等项目建设，新增生猪规模养殖200万头，三年内突破1000万头。强化农业科技服务，加大农畜新品种新技术推广。围绕三次产业融合培育农业新型生产经营主体。巩固发展种养大户、家庭农场和合作社，促进规模化生产主体培育，新增一体化合作社20个；发挥农业公司联接基地、对接市场、经营灵活的优势，拓展个性化需求市场，推动农副产品加工营销主体培育，选树农业示范公司20个；以“粮头食尾”“农头工尾”为抓手，加快农产品精深加工战略龙头企业培育。加快阜丰300万吨玉米深加工、飞鹤奶山羊养殖及乳品加工、嘉一香生猪屠宰加工等项目建设，推动益海嘉里粮食综合加工等项目开工建设。加快生猪精深加工项目布局，延展生猪产业链。推进国家级现代农业示范园创建。围绕价值链加大农产品营销。主打生态原产地、非转基因产品，培育“鹤城”等地域品牌。加快发展农产品电商，每县至少培育一个地域品牌和自建一个电商平台，利用大米网、东北黑土网等营销平台，提高农产品线上销售量。锁定目标城市，强化区域集中推广营销合作，以广州、南京为切入点，加大珠三角、长三角地区农产品销售。围绕释放活力深化农村重点改革。完善承包地“三权”分置制度，完成土地确权登记颁证。加快清产核资，推进农村集体产权制度改革。改进耕地占补平衡管理办法。深化供销社综合改革，提升服务“三农”能力。健全自治、法治、德治相结合的乡村治理体系，探索乡村治理新模式。

　　（二）以建设“中国制造2025”哈大齐国家级示范区为牵动，着力打造具有核心竞争力产业集群，加快建设工业强市。围绕12个重点产业和倍增计划，以先进装备制造和食品加工为主体、其他重点产业为补充，老企业改造升级和新项目引进建设并重，进一步释放潜量、盘活闲量、做优存量、引进增量，加快构建“2+X”新型制造业体系。争取用三年时间，打造一批百亿级重点企业，建设装备、食品两大千亿级产业集群。深入实施“五化”示范工程。重点推进一重军工、北方高科稀土合金等产品高端化；推进齐重数控智能制造基地、黑龙冰刀数字化车间等工艺智能化；推进紫金铜产业园、大力矩节能电机等生产绿色化；推进齐重数控产品“全生命周期管理”、华工机床洁净钢共性技术服务平台等业态服务化；推进一重、齐车、北钢、景宏石油等合作国际化。大力实施规上企业助力工程。重点在企业直供电、物流配套、专项奖补资金、贷款周转金、助保金贷款等方面搞好服务，深化涉企收费改革，促进企业降本增效。建立健全服务、引领、激励长效机制，引导规上企业增强核心竞争力。加快实施中小企业培育工程。重点围绕骨干企业和新上大项目，大力发展吃配经济，促进产业链延伸。发挥中小企业服务中心作用，在成长型企业项目申报、融资担保、要素保障等方面给予重点扶持。深入开展“保规”行动，新增规上企业30户以上。

　　（三）以强化电商融合带动为途径，着力发展新业态新模式，加快推进服务业现代化。推动商贸业态融合发展。推进电商与实体商贸业深度融合，进一步提升域内、贯通域外、拓展境外市场。通过多层次培训、多方式触网、多渠道创业、多元化运营、专业化推进，全力推动电商务实发展。全年新增电商企业100户、网店1.2万家，培育精品微商社群10个，上行交易额增长30%以上；限上企业触网率超过70%。加快发展多业态新型商贸综合体，推进物流服务和市场流通体系融合发展，新增限上企业30户。推动旅游业一体化服务发展。围绕“鹤城”生态特色，加快建设旅游强市。以扎龙、明月岛和旅游小镇等为重点，坚持产品化打造景区、市场化经营景区、综合业态提升景区、线上线下一体化拓展景区，培育品牌旅游目的地；围绕吃住行游购娱各环节，开展创优达标活动，培育旅游产业服务链；围绕重点景区和域内精品线路，培育旅行社、旅游电商等推广营销主体，推进线上线下一体化服务。加强旅游集中推广和市场监管，促进旅游业健康发展。接待游客和旅游收入分别增长10%以上。推动金融与实体经济协同发展。促进政银企保深度合作，创新金融产品。加快“社改行”进程，积极引进直接融资类金融机构。完善担保体系建设。发展服务金融的专业律师和会计师事务所，引导企业进入多层次资本市场，推动飞鹤乳业、恒阳牛业上市。强化地方金融监管，有效防范和化解金融风险。推动冰雪体育运动产业快速发展。深入开展“百万人上冰雪、百万人游鹤城”活动，办好冰球节和冬夏两个冰球季，叫响冰球城市品牌。着眼于竞技体育产业化、群众体育社会化、体育运动装备制造规模化，培育体育俱乐部、赛事公司、培训基地、各类协会等产业主体，大力发展赛事经济。推进黑龙冰刀滑雪板等项目建设。全年建设冰场180处，新组建冰球队30支以上，举办赛事活动200项（次）以上。推动养老产业多元发展。完善居家养老社会化服务体系，加快发展医养结合式特色养老，拓展生态候鸟式养老，适度扩大中高端养老，探索城乡困难群体养老模式，满足多层次养老需要。全年新增养老床位2000张以上。推动文化产业创新发展。加快公办演出单位和场所市场化改革，培育和引进文化产业主体，推动本地工艺美术、工艺陶瓷产业化，促进文化产业持续发展。

　　（四）以创建哈大齐国家自主创新示范区为目标，着力培育科技型产业主体，加快创新驱动发展。把创新作为第一动力，全力打造全面振兴发展新动能。加快技术创新体系建设。支持G7联盟、燕大产业研究院、哈工大产业研究分院、齐大食品研究院、齐医黄芪产业研究院及各类驻齐科研机构与域内企业对接合作，加快本地科技成果生成和域外科技成果引进，建立科研成果归集和推广平台，全年生成科技成果260项，引进落地40项。发挥高新区创新龙头作用，加快构建以“孵化器-加速器-园区”为链条的科研成果产业化体系，带动全市各类园区科研成果产业化。高新区孵化器入驻项目80个以上。引导企业技术中心、工程技术研究中心、院士工作站以及重点实验室服务地方经济发展，引导企业与科研机构组建产学研创新联盟，构建以企业为主体、市场为导向、产学研深度融合的技术创新体系。完善政策、资金等技术创新服务体系。新增国家高新技术企业6户，培育一批科技型企业，加快形成科技创新产业集群。推动重点群体创新创业。鼓励科技人员领办创办科技型企业，扶持大学生、下岗职工和农民自主创业。推动技能培训基地向创业平台转型，全市创业平台达到240个，新建大学生实习见习基地200个以上。弘扬劳模精神和工匠精神，举办各类创新创业大赛和劳动技能大赛，落实技能提升补贴政策，营造创新创业氛围。强化人才支撑。落实各项政策措施，坚持岗位和待遇留人，培育造就一批满足发展需要的人才和创新团队。实施“企业家发展计划”专项行动，分行业、分领域搭建服务企业家平台，建立学习培训、交流合作、评价激励机制，把企业家凝聚起来创新创业。推进名校优生引才工程。加强人才库建设，搭建高端人才聚集平台，提升人才服务水平。

　　（五）以深化经济体制改革为重点，着力优化发展环境，加快释放市场主体活力。深化“放管服”改革。以“减”为目标，深入清理规范行政权力事项。全面推行“多规合一”“多审合一”“一表一窗联审”“承诺制、零审批”投资审批制度。深化“多证合一”“证照分离”等商事制度改革，加强事中事后监管。以为民服务事项为重点，深化行政审批和完善“一窗式”政务服务改革。深化综合执法体制改革，加快执法重心下移。全面推行执法公示制度、执法全过程记录制度和执法决定法制审核制度，深入推行“双随机、一公开”执法机制。建立守信激励和失信惩戒机制，完成市级信用平台基础建设。落实市场准入负面清单制度，营造公平竞争环境，支持民营企业发展。深化产权制度改革。完善各类国有资产管理体制，改革国有资本授权经营体制，分领域建立国有资产运营公司。支持中省属企业改革，分类推进市属国企产权制度改革。深化要素市场化改革。深化公共资源交易体制改革，完善审批、监督、实施相分离工作机制，推进交易全流程电子化。按照中央和省统一部署，有效落实财税、金融、电力、价格等重大改革政策和举措。

　　（六）以解决发展不平衡为导向，着力优化经济布局，加快区域协调发展。加快开放发展。抢抓国家实施“一带一路”战略机遇，强化国际经贸交流合作，努力打造面向俄欧、东北亚全面开放新格局。完善互联互通基础设施，建成进口肉类指定查验场，积极申报保税功能区和临时铁路口岸，加快三间房国际经贸物流产业园建设。加大外向型企业培育，扶持一重、齐车、北钢、北欧卫浴等企业扩大进出口加工，推进梅里斯中俄优质农产品交互性贸易周转中心等项目建设，发展跨境电商新业态，打造对外开放生力军。新增进出口企业20户。强化城乡协调发展。发挥城区经济龙头作用和县域经济基础作用，形成协调发展、两翼齐飞的良好格局。坚持市区联动，城区经济在巩固提升装备制造业的同时，大力发展现代服务业。把发展壮大县域经济的突破口放在食品工业上，推动县域经济晋位赶超。同时，以中心城区为引领，以县域城关镇为主体，带动乡镇协调发展，加快全域城镇化步伐，加快农业转移人口市民化。推动合作发展。用足用好国家政策，在加快东北等老工业基地振兴战略中争取更多发展机会。深入推进我市与广州对口合作，在各领域实现互惠共赢。主动对接京津冀协调发展，承接好符合我市发展方向的重点产业。促进地企协同发展。加快国有企业“三供一业”移交，支持央企产业升级和人才培养，携手推动央企做大做强。推广一重地企合作发展模式，扩大企业项目和技术溢出，提高地方产业合作配套。注重军民融合发展。充分发挥军工科研和生产优势，重点在军转民、民参军上实现突破，加快碾子山军工旅游、红旭达雷达设备列装等项目建设。突出园区建设集约发展。围绕12个重点产业、县区主导产业,加快各类产业园区的龙头项目、配套项目、服务体系建设，以高质量项目提升区域经济发展规模、质量和效益。

　　二、坚持以人民为中心，深入实施“七大民生工程”，不断增强人民群众的获得感幸福感

　　坚持尽力而为、量力而行，深入实施“七大民生工程”，让改革发展成果更多更公平惠及全市人民。

　　（一）深入实施脱贫攻坚工程。坚持精准扶贫精准脱贫，主攻产业扶贫软肋，提高产业扶贫覆盖率和实效。注重补齐短板，强化“两不愁、三保障”质量。加大资金整合投入力度，金融扶贫贷款规模达到10亿元。加强考核监督和全过程跟踪审计，做到脱真贫、真脱贫。坚持扶志、扶智相结合，激发贫困群众内生动力。完成132个贫困村、5.3万贫困人口脱贫任务，力争3个县脱贫摘帽，打好精准脱贫攻坚战。

　　（二）深入实施社会保障工程。开展就业服务行动。继续实施援企稳岗政策，大力开发新就业岗位，实现城镇新增就业再就业8.6万人。开展扩面提待行动。城乡居民养老保险覆盖面超过150万人；落实养老金提标政策，确保按时足额发放。城乡居民医保最高支付限额再提高2万元，扩大医保药品使用范围，降低贫困人群大病医保起付线，特殊门诊慢性病受益范围扩至城乡居民医保全部人群。稳步提高失业保险待遇。开展社会救助行动。强化精准救助，按政策提高城乡低保等救助标准。发展老龄事业，提升养老院服务质量。加快实施残疾人小康进程规划，残疾人康复中心投入使用。抓好“双拥”和优抚工作。提升防灾减灾救灾能力。

　　（三）深入实施宜居工程。开展保障房改造行动。全市棚户区改造竣工1.6万户，开工建设公租房1440套，改造农村贫困户危房5000户。开展老旧小区综合改造行动。以既有建筑节能改造、“三水”改造、小区环境综合整治为重点，改造老旧小区10个。开展保供行动。启动铁路大水源等扩建工程，完成浏园水厂新建水源地、浏园水厂二期等工程，改善城镇供水质量。加快集中供热热源建设，强化热源热网平衡，全力提升供热质量。开展物业服务提升行动。强化服务标准，开展物业企业信用等级评定，提升物业服务质量。探索物业服务多元化机制，解决老旧单散小区物业难题。开展住房为民行动。增加优质商品房有效供给，加大去库存力度，规范市场行为，加快建立多主体供应、多渠道保障、租购并举的住房制度，促进房地产市场平稳健康发展。

　　（四）深入实施城市交通改善工程。开展路网优化行动。完善支路规划，逐步畅通道路微循环。推进中心城区21条道路改扩建，完成12个路口渠化，完成南外环建设，谋划民航路跨线桥建设。开展停车规范行动。优化停车场布局，加强学校、商场和农贸市场等重点区域停车管理，施划公共停车位1万个；强化住宅小区停车管理，落实租售并举制度，推进复合式停车场建设，施划小区停车位1万个。开展公交升级行动。完成卜奎大街、安顺路等公交专用道设置，更换新能源公交车100台，优化公交站点和线路，改善公交乘车环境，强化服务标准，提高服务水平。开展管理提效行动。优化交通组织，规范交通行为，加强标识、信号、隔离和监控系统等基础建设，提高道路畅通率。

　　（五）深入实施城市环境整治工程。持续开展“四季环境整治”，进一步优化城市人居环境。开展全民绿化行动。广泛动员全社会参与，完成中心城区37条道路、15个小区、8个公园游园绿化提升工程，栽植树木45万株。开展全城亮化行动。坚持谁所有谁负责、应亮尽亮、简约实用，抓好沿主干路、沿江、沿湖等主要楼体和大型设施亮化，完善公交站点和桥梁、广场、公园绿地亮化。开展清洁行动。全面落实网格制清扫清运保洁责任，整治垃圾死角，完善环卫设备设施，提高环卫水平。推进固体废弃物静脉产业园建设和红星垃圾场搬迁治理，全市生活垃圾无害化处理率达到85%以上。开展市容整治行动。集中清理整治城市“八乱”现象，建立网格式长效管控责任机制，促进市容环境水平提升。

　　（六）深入实施社会事业提升工程。开展教育为民行动。改扩建幼儿园15所，改造薄弱学校60所，建设义务教育标准化学校32所，确保8个县（市）区通过国家义务教育基本均衡县（区）验收，推动教育优质均衡发展。从群众最不满意的事情改起，开展丰富多彩的校园课外活动，延伸优化课后服务；深化中高考改革，促进素质教育提升；规范发展幼儿教育；着力解决教育不公问题；推动教师队伍管理改革，加强师德师风建设，狠刹教师有偿补课歪风；推动“双元培育”职业教育改革，促进产教融合。开展健康鹤城行动。深入推进公立医院改革，扎实推动医联体、医共体建设，完善基层服务体系，多渠道推进家庭医生签约并做实服务，加大全科医生培养力度，推广名医工作室，大力发展中医药事业，有效缓解看病难看病贵问题。广泛开展全民健身活动。开展文化惠民行动。办好“四季鹤城”等品牌文化活动，惠民演出260场。支持群众性文化活动。多渠道筹措资金，推进卜奎清真寺、“516”遗址、江桥抗战遗址等重点文物修缮、保护和利用。

　　（七）启动实施农村人居环境整治工程。以中心屯为重点，以中心村为带动，梯次推进村屯全面整治。推进农村改路、改水、改厕、改灶、改圈。建设农村公路300公里，解决733个村屯、1.75万贫困人口饮水安全问题。推进农村“五乱”现象整治，解决村屯环境突出问题。推进供水、供电、垃圾清运及保洁，提升农村公共服务水平。推进村屯绿化美化，营造良好环境。结合美丽乡村建设，开展“文明家庭”等评选活动，带动农民素质提升。

　　三、坚持人与自然和谐共生，加快推进生态文明建设，努力打造美丽鹤城

　　坚持节约优先、保护优先、自然恢复为主，着力释放生态优势，加快建设生态强市。

　　（一）推进绿色发展。践行绿色生产方式，引导企业节能降耗，推进土地、矿产等资源节约有效利用。推进节水行动，严守水资源管理“三条红线”。争创国家可再生能源综合应用示范区，壮大节能环保产业规模，推进清洁低碳和循环经济发展。通过兼并重组、破产清算等方式清理僵尸企业，释放闲置无效资源。倡导简约适度、绿色低碳的生活方式，开展节约型机关、绿色家庭、绿色学校、绿色社区和绿色出行等创建活动。在做好节约保护的基础上，发展生态经济，促进生态优势向经济优势转变。

　　（二）打好污染防治攻坚战。深入开展“三大防治”行动，尤其要打好蓝天保卫战，全年空气质量优良天数达到86%以上；深入实施“水十条”，地表水Ⅲ类以上水体比例达到66%以上；全面实施“土十条”，土壤污染持续下降。重点实施9项专项治理：实施燃煤污染专项治理，配齐燃煤锅炉脱硫脱硝除尘设施，污染物稳定达标排放。实施秸秆污染专项治理，严控秸秆焚烧，市场化推进秸秆肥料化、饲料化、燃料化、原料化、基料化综合利用。实施扬尘污染专项治理，落实粉状物料“储、运、用”控制措施，提高机械湿扫率。实施移动污染源专项治理，加强机动车尾气遥感监测和环保检测管理，加大黄标车及老旧车辆淘汰力度。实施城市生活污水专项治理，加快城区污水系统改造和城市黑臭水体整治，推进县级污水收集和处理设施提标改造。实施工业污水专项治理，严格企业废水排放标准，建立违法企业黑名单，落实企业环保信息公开、信用评价制度。实施畜禽粪污专项治理，强化畜禽养殖废弃物资源化利用，畜禽粪污处理设施配套率和资源化综合利用率分别达到75%和55%以上。实施工业企业土壤污染专项治理，落实企业环境保护主体责任和常态化管理制度，科学处置工业固体废弃物。实施农业土壤污染专项治理，持续推进农业“三减”，提高农田废旧地膜清理率，防治农业面源污染。

　　（三）加大生态保护力度。坚持现状保护和生态修复相结合，全面完成生态“三条控制线”划定工作。强化水生态保护。全面落实各级河长责任制，加强流域综合治理，促进水生态修复。强化山水林田湖草系统治理，维护河湖生态功能。启动劳动湖生态修复、中心城区沿江生态整治工程。强化湿地生态保护。深入开展“绿盾行动”，加强湿地修复与保护，扎实推进扎龙和乌裕尔河、双阳河等自然保护区问题整治。强化森林生态保护。扎实推进国土绿化行动，加快完善三北防护林工程，植树造林6.5万亩。强化草原生态保护。加快草原生态治理，深化退牧还草工程建设，新增人工牧草3万亩、改良草原1万亩。强化土壤生态保护。推进土壤资源化利用，严格保护耕地，深入推行耕地科学轮作。抓好龙江、克山黑土地保护利用试点。治理水土流失15万亩。

　　（四）改革生态监管体制。加快生态环境大数据平台建设。优化国土空间开发格局，健全国土资源节约集约利用机制。坚持总量平衡原则，实施自然资源资产双量管控机制。制定最严格的环境保护措施，完善生态文明考核评价体系和环境监管体系，积极推进自然资源资产责任审计，严格落实生态环境损害责任追究制度，严厉打击破坏生态环境行为。

　　四、坚持打造共建共治共享社会治理格局，持续深化“平安鹤城”建设，不断加强和创新社会治理

　　完善党委领导、政府负责、社会协同、公众参与、法治保障的社会治理体制，提高社会治理能力和水平。

　　（一）加强社会治安防控体系建设。持续推进公安数据警务、基层基础、规范执法、治安防控、预警信息、素质能力“六大工程”建设，健全打防管控工作机制，深入开展“命案必破”“大案攻坚”“黄赌毒”“黑拐骗”“传销”“信息犯罪”等严打行动，深化治安大巡防和“一村一辅警”建设。强化治安要素源头管控，最大限度消除治安隐患和管理盲区，切实增强群众安全感。

　　（二）加强信访维稳体系建设。严格执行重大事项、重大决策风险评估制度，加强社会稳定风险隐患排查。严格落实信访工作责任制、领导干部接访下访、重大疑难问题会商、统筹督查督办等制度。畅通信访渠道，推动积案化解和依法分类处理，提升信访工作专业化、法治化、信息化水平，努力解决群众合理合法利益诉求。

　　（三）加强安全生产体系建设。牢固树立“隐患就是事故”理念，全面落实“党政同责、一岗双责、齐抓共管、失职追责”和“三个必须”要求。强化企业主体责任，落实安全生产和职业健康工作职责。完善安全风险防控和隐患排查治理机制，构建双重预防体系。加强监管责任落实，强化过程监督，开展专项整治，推动隐患整改，有效预防和减少事故，确保群众生命财产安全。

　　（四）加强市场监管体系建设。完善食药品安全监管体系，落实“属地管理、部门监管和企业主体”责任，推行食品安全追溯机制，开展学校食堂、校园周边、养老机构、大型餐饮、网络订餐等重点领域专项整治行动。加强市场监管，严把质量关口，依法打击制假售假侵权等行为，保护生产者和消费者权益。

　　（五）加强社区治理体系建设。加强社区班子、社工队伍和服务体系建设，提高社区自我管理、自我服务水平。充分发挥社区联系群众、服务群众桥梁纽带作用，做好法律援助、社区矫正、人民调解、安置帮教等工作，强化社区治理能力。新增城乡示范社区15个。城乡社区综合服务设施覆盖率分别达到80%和30%。同时，规范管理社会组织，加强民族宗教工作。

　　五、坚持全面从严治政，加强政府自身建设，努力建设人民满意的服务型政府

　　贯彻落实好党的十九大精神，适应新时代发展要求，政府系统必须始终坚持党的领导，加强政治建设，转变行政方式，提高服务本领。

　　（一）切实增强政治自觉。坚持用习近平新时代中国特色社会主义思想武装头脑，把政治意识、大局意识、核心意识、看齐意识贯穿政府工作始终，坚定执行党的各项决策部署，坚决维护以习近平同志为核心的党中央权威和集中统一领导。深入推进“两学一做”学习教育常态化制度化，扎实开展“不忘初心、牢记使命”主题教育，加强党性锻炼，坚定理想信念，始终对党忠诚、对领袖忠诚、对人民忠诚。

　　（二）推进法治政府建设。深入贯彻全面依法治国总要求，带头尊法学法守法用法，坚决维护法律权威。推行政府部门法律顾问制度，实行重大决策合法性审查。依法完善权力事项标准，建立权责清单,规范权力运行。认真办理人大议案、政协提案，广泛听取意见建议，自觉接受人大监督、政协监督、社会监督和舆论监督。科学做好地方性法规草案审修，严格审查政府规章和规范性文件。深入推进政务公开。

　　（三）提升政府施政能力。注重转方式，加强学习和实践，强化市场化工作思维，培养专业能力，弘扬专业精神，促进政府系统由注重体制内推动经济向服务激发市场主体活力转变、由传统模式推动工作向以改革创新引领现代化发展转变。以精简高效为目标，深化机构改革，分类推进事业单位改革。注重提效能，践行“四零”服务承诺，完成政务云平台建设，推进市域政务协同办理一体化，实现“一号申请、一窗受理、一网办结、一城通办”，让“信息跑路”代替“群众跑腿”，打好重要领域改革攻坚战。注重抓落实，完善执行、监督、考评、奖惩机制，强化重点工作全过程闭环督查督办，建立健全以大事项为引领、推进各项工作落实的长效机制。

　　（四）持之以恒正风肃纪。深入贯彻全面从严治党新要求，坚决落实党风廉政建设责任制和“一岗双责”，深入推进战线负责制，层层压实责任。强化教育警示和制度规范，把权力置于阳光下运行，从源头上预防腐败。坚持敬畏权力、慎独慎微、干净干事，着力构建清爽同志、亲清新型政商关系。坚决落实中央八项规定实施细则，严防严查形式主义、官僚主义新表现，打好整顿作风优化发展环境攻坚战。牢固树立过紧日子思想，立足保基本、保民生，强化财政四本预算和专项预算管控，全面实施财政绩效管理。严控机关事业单位编制使用，人员编制只减不增。严控“三公经费”和楼堂馆所建设，坚决反对形象工程。严控规费标准，杜绝弹性征管，避免权力寻租。严格规范各类政府债务“借管用还”行为，有效防范和化解债务风险。全力支持各级纪委和监察委工作，发挥审计监督作用，严查破坏发展环境行为，严惩不正之风和腐败问题。

　　各位代表，进入新时代，站在新起点，开启新征程，需要我们使命担当、改革创新、真抓实干。让我们更加紧密地团结在以习近平同志为核心的党中央周围，在省委、省政府和市委的坚强领导下，不忘初心，牢记使命，锐意进取，为决胜全面建成小康社会、建设富强民主文明和谐美丽新鹤城而不懈奋斗！
